# Supplementary material for: Current Challenges in the Treatment of the Omphalocele—Experience of a Tertiary Center from Romania
Source: J Clin Med. 2022 Sep 27;11(19):5711. doi: 10.3390/jcm11195711 (PMC9573750; doi:10.3390/jcm11195711)
Supplement: Supplementary file 1 [file jcm-11-05711-s001.zip › jcm-1900413-supplementary.pdf]

Supplementary materials

Table S1. Descriptive statistics of the patients

Indicator	DECADE	N	Mean	Median	Min	Max	Shapiro-Wilk	
							W	p
GESTATIONAL AGE (weeks)	1	46	37.96	39.00	30.00	43.00	0.79	< .001
	2	53	36.32	38.00	29.00	41.00	0.90	< .001
	3	40	36.95	38.00	27.00	40.00	0.74	< .001
	Overall	139	37.04	38.00	27.00	43.00	0.85	< .001
BIRTH WEIGHT (grams)	1	46	2746.96	2750.00	1700.00	4700.00	0.93	0.009
	2	53	2665.09	2700.00	900.00	4200.00	0.99	0.824
	3	40	2764.25	2900.00	940.00	4100.00	0.94	0.024
	Overall	139	2720.72	2750.00	900.00	4700.00	0.99	0.532
LENGHT (centimeters)	1	46	47.98	49.00	37.00	53.00	0.90	0.001
	2	53	46.93	48.00	31.00	54.00	0.93	0.003
	3	40	47.56	48.00	35.00	52.00	0.87	< .001
	Overall	139	47.46	48.00	31.00	54.00	0.90	< .001
DEFECT SIZE (centimeters)	1	46	6.85	6.00	2.00	18.00	0.91	0.002
	2	53	6.83	6.00	3.00	18.00	0.87	< .001
	3	40	5.50	5.00	2.00	15.00	0.86	< .001
	Overall	139	6.45	5.50	2.00	18.00	0.89	< .001
LENGTH OF HOSPITALIZA TION (days)	1	46	16.81	10.00	1.00	54.00	0.85	< .001
	2	53	24.69	14.00	1.00	121.00	0.71	< .001
	3	34	21.18	12.50	2.00	153.00	0.53	< .001
	Overall	133	21.07	12.00	1.00	153.00	0.68	< .001
AGE AT THE TIME OF SURGERY (hours)	1	26	51.69	18.00	3.00	316.00	0.64	< .001
	2	29	48.19	12.00	3.00	252.00	0.68	< .001
	3	29	75.38	40.00	6.00	432.00	0.71	< .001
	Overall	84	58.66	20.50	3.00	432.00	0.69	< .001
AGE AT DEATH (days)	1	24	9.21	5.40	0.50	54.00	0.65	< .001
	2	31	13.98	7.00	1.00	81.00	0.74	< .001
	3	17	25.96	10.00	2.00	153.00	0.59	< .001
	Overall	72	15.22	6.90	0.50	153.00	0.56	< .001
AGE AT ADMISSION (hours)	1	46	10.95	5.00	0.50	102.00	0.46	< .001
	2	53	10.34	4.50	0.50	160.00	0.34	< .001
	3	39	12.56	5.50	1.50	84.00	0.55	< .001
	Overall	138	11.17	5.00	0.50	160.00	0.44	< .001
MOTHER'S AGE (years)	1	46	27.30	26.00	17.00	43.00	0.95	0.039
	2	53	26.19	25.00	15.00	45.00	0.93	0.005
	3	38	30.18	31.00	17.00	45.00	0.97	0.364
	Overall	137	27.67	27.00	15.00	45.00	0.96	< .001
FATHER'S AGE (years)	1	46	31.04	30.00	21.00	49.00	0.94	0.029
	2	53	30.34	30.00	18.00	48.00	0.97	0.180
	3	37	35.62	37.00	22.00	49.00	0.97	0.341
	Overall	136	32.01	31.00	18.00	49.00	0.97	0.009

Table S2. Model summary

Step	-2 Log likelihood	Cox & Snell R Square	Nagelkerke R Square
1	165.685	0.175	0.233
2	144.466	0.291	0.389
3	132.079	0.352	0.469
4	121.620	0.399	0.532
5	113.630	0.432	0.577
6	108.090	0.455	0.607

Step 1. Predictors: (Constant), Sepsis

Step 2. Predictors: (Constant), Sepsis, Acute renal failure

Step. 3 Predictors: (Constant), Sepsis, Acute renal failure, Associated abnormalities

Step. 4 Predictors: (Constant), Sepsis, Acute renal failure, Associated abnormalities, Hemorrhagic disease

Step. 5 Predictors: (Constant), Sepsis, Acute renal failure, Associated abnormalities, Hemorrhagic disease, Treatment

Step. 6 Predictors: (Constant), Sepsis, Acute renal failure, Associated abnormalities, Hemorrhagic disease, Treatment, Prematurity

Table S3. Variables in the Equation

Variables		B	S.E.	Wald	df	Sig.	Exp(B)	95% C.I.for EXP(B)	
								Lower	Upper
Step 1[a]	Sepsis (1)	2.063	0.444	21.595	1	0.000	7.871	3.297	18.792
	Constant	-0.505	0.214	5.569	1	0.018	0.603		
Step 2[b]	Sepsis (1)	1.936	0.470	16.937	1	0.000	6.933	2.757	17.433
	Acute renal failure (1)	2.784	0.780	12.746	1	0.000	16.176	3.509	74.566
	Constant	-0.867	0.243	12.723	1	0.000	0.420		
Step 3[c]	Sepsis (1)	2.246	0.508	19.517	1	0.000	9.449	3.489	25.594
	Associated abnormalities (1)	1.598	0.473	11.426	1	0.001	4.942	1.957	12.479
	Acute renal failure (1)	2.538	0.802	10.021	1	0.002	12.655	2.629	60.910
	Constant	-1.468	0.328	19.986	1	0.000	0.230		
Step 4[d]	Sepsis (1)	2.288	0.545	17.648	1	0.000	9.854	3.389	28.654
	Associated abnormalities (1)	1.711	0.510	11.235	1	0.001	5.534	2.035	15.050
	Acute renal failure (1)	2.035	0.816	6.222	1	0.013	7.656	1.547	37.896
	Associated abnormalities (1)	2.162	0.746	8.405	1	0.004	8.689	2.014	37.476
	Constant	-1.781	0.373	22.771	1	0.000	0.169		
Step 5[e]	Sepsis (1)	2.286	0.565	16.357	1	0.000	9.835	3.248	29.779
	Associated abnormalities (1)	1.907	0.545	12.229	1	0.000	6.730	2.312	19.594
	Acute renal failure (1)	2.274	0.858	7.019	1	0.008	9.721	1.807	52.292
	Treatment (1)	1.342	0.493	7.416	1	0.006	3.828	1.457	10.057
	Hemorrhagic disease (1)	2.079	0.773	7.229	1	0.007	7.994	1.757	36.380
	Constant	-2.452	0.484	25.708	1	0.000	0.086		

Step 6[f]	Sepsis (1)	2.567	0.604	18.053	1	0.000	13.031	3.987	42.589
	Associated abnormalities (1)	1.837	0.566	10.532	1	0.001	6.276	2.070	19.029
	Acute renal failure (1)	2.382	0.885	7.243	1	0.007	10.824	1.910	61.340
	Treatment (1)	1.330	0.509	6.814	1	0.009	3.780	1.393	10.257
	Hemorrhagic disease (1)	1.712	0.753	5.166	1	0.023	5.543	1.266	24.267
	Prematurity (1)	1.286	0.557	5.323	1	0.021	3.618	1.213	10.789
	Constant	-2.858	0.558	26.192	1	0.000	0.057		

a. Variable(s) entered on step 1: Sepsis

b. Variable(s) entered on step 2: Acute renal failure

c. Variable(s) entered on step 3: Associated abnormalities

d. Variable(s) entered on step 4: Hemorrhagic disease

e. Variable(s) entered on step 5: Treatment

f. Variable(s) entered on step 6: Prematurity

Table S4. Confusion matrix

**Observed**	**Predicted**		**Correct**
	**NO**	**YES**	
NO	56	10	84.848
YES	11	62	84.932
**Overall % Correct**			**84.892**

Table S5. Collinearity Statistics

**Predictor**	**Tolerance**	**VIF**
Sepsis	0.783	1.278
Acute renal failure	0.949	1.053
Associated abnormalities	0.802	1.246
Hemorrhagic disease	0.956	1.046
Treatment	0.901	1.110
Prematurity	0.883	1.133

VIF = Variance inflation factor
